# The Introduction of Thousands of Tonnes of Glyphosate in the food Chain—An Evaluation of Glyphosate Tolerant Soybeans

**DOI:** 10.3390/foods8120669

**Published:** 2019-12-11

**Authors:** Thomas Bøhn, Erik Millstone

**Affiliations:** 1 Institute of Marine Research, 9006 Tromsø, Norway; 2 Science Policy Research Unit, University of Sussex, Brighton BN1 9SL, UK; e.p.millstone@sussex.ac.uk

**Keywords:** *Daphnia magna* feeding studies, food chain, genetically modified organism (GMO) safety testing, glyphosate residues in soybean, health risk, risk assessment, resistance evolution, Roundup, toxicity

## Abstract

Glyphosate-tolerant (GT) soybeans dominate the world soybean market. These plants have triggered increased use of, as well as increased residues of, glyphosate in soybean products. We present data that show farmers have doubled their glyphosate applications per season (from two to four) and that residues of late season spraying of glyphosate (at full bloom of the plant) result in much higher residues in the harvested plants and products. GT soybeans produced on commercial farms in the USA, Brazil and Argentina accumulate in total an estimated 2500–10,000 metric tonnes of glyphosate per year, which enter global food chains. We also review studies that have compared the quality of GT soybeans with conventional and organic soybeans. Feeding studies in *Daphnia magna* have shown dose-related adverse effects (mortality, reduced fecundity and delayed reproduction) of glyphosate residues in soybeans, even at glyphosate concentrations below allowed residue levels. We argue that GT soybeans need to be tested in fully representative and realistic contexts. However, the current risk assessment system has only required and received data from field trials with beans that were sprayed with much lower doses of glyphosate as compared to contemporary commercial farms. This has left knowledge gaps and a potentially serious underestimation of health risks to consumers.

## 1. A Quantitative Success

Glyphosate tolerant (GT) soy deserves sustained attention from researchers and regulatory authorities because several safety issues have not yet been resolved. It is the dominant genetically modified (GM) plant and trait combination on the global market. About 77% of the global soybean production comes from GT soybean and the dominant soy producing countries of Brazil, USA and Argentina have a 94%–100% adoption rate of ‘biotech crops’, mostly glyphosate tolerant varieties [1]. This development in agro-industrial technology has been reported to have contributed to increased gross farm incomes mainly by reducing production costs [2]. 

GT soybean agriculture is a ‘technological package’—a plant-and-herbicide-combination—which enables farmers to kill weeds by spraying herbicides during the growing season, except those weeds that have developed tolerance to glyphosate. The genetic modification makes the soy plants tolerate the herbicide. Commercially, the rapid growth in sales and use of glyphosate-based herbicides (GBHs) such as Roundup has been linked to the success of the glyphosate tolerant soybean seeds [3]. As 349 million metric tonnes (MT) of soy were produced in the 2016–2017 season [4], out of which GT varieties contributed about 270 million MT, it is important to test potential quality differences between GT soybeans and: (i) conventional agro-industrial soybeans (to which herbicides are only applied outside the growing season and onto undesirable plants) and (ii) organic soybeans (to which herbicides are not applied). Moreover, the agricultural practice that was initiated by the introduction of GT GM plants in 1995, in which herbicides are sprayed directly on the plants during the growing season, makes the issue of glyphosate residues in the food chain increasingly relevant.

## 2. A Qualitative Evaluation

Globally, soybean is a dominant livestock feed ingredient, potentially affecting very large numbers of consumers of plant material as well as meat eaters and thus should be subject to intense scrutiny from leading agricultural and food safety authorities, such as the US Food and Drug Administration (FDA), the European Food Safety Authority (EFSA), the World Health Organisation (WHO) and from independent researchers. Studies able to inform risk assessments can investigate, for example, the characteristics and consequences of different methods of cultivation, the genetic transformation, new proteins, potential allergens, nutritional composition and other potential adverse effects on public and/or environmental health. 

Interestingly, despite the substantial quantities of published scientific evidence, the characteristics and implications of GM plants have been long contested. Although the US FDA has claimed that such GM plants have been produced with precision and so increase the chance of providing more predictable and safer foods [5], numerous research findings have contradicted that claim, revealing amongst other things that the technology can be imprecise and its consequences hard or impossible reliably to predict. For example, the locations of inserted genes can be random, and the resulting plants may have altered, activated or inactivated endogenous genes, as well as the intended changes [6]. Random and unintended integration of transgenes into recipient plant genomes present potentially problematic biosafety issues [7] although a majority of the safety studies of GM plants have so far concluded that those plants can be as safe and nutritious as conventional plants [8]. However, as Domingo and Bordonaba have noted, most of the studies claiming safety of GM products were conducted by the biotechnology companies that are responsible of commercializing those novel varieties [9]. This is in line with previously described problems in regulatory safety assessments related to the concept of ‘substantial equivalence’ [10]. A review that analysed 94 articles on the putative health risks of GM products, showed that the selection of the study outcomes reported in individual papers were significantly influenced by financial and/or professional conflicts of interest [11]. The scientific and policy controversies over GM foods are still important and remaining uncertainties about chronic, as opposed to acute risks, can not be solved as long as studies of long-term health effects (i.e., more than 90 days) are still not required [9]. Moreover, some independent researchers have even suffered personal attacks as well as with threats of legal action from companies trying to prevent the publication of critical results on GM crops [12,13]. 

In the most commonly used GT soybean plant, based on the GTS 40-3-2 event (first generation Roundup Ready), evidence shows that a large 250 kb fragment of the inserted transgene is also present in a repeated, second copy located downstream of the main insertion in the genome. In 2005, it emerged that a majority of this fragment is transcribed and further processed, illustrating how new unknown fusion proteins may be produced by this and other GM plants [14]. Such unanticipated alterations should be thoroughly investigated in a fresh risk assessment, but that has yet to be accomplished. However, data that reveal marked increases in the quantities of glyphosate residues in soybeans intended for the food-chain may potentially be more important in relation to public health. At current rates of use, glyphosate has become widely present in the global soybean supply and the pragmatic regulatory response has been to accommodate those changes in glyphosate use by markedly increasing levels of daily intakes deemed to be tolerable [15]. Although glyphosate-resistant soybeans are presently not grown in Europe, their widespread cultivation in the USA and Latin America has resulted in substantial residues of glyphosate in soy products imported into Europe for food and feed [16].

In order to furnish evidence that might diminish the controversy, one of the authors of this article (Bøhn) initiated a four-year research project to investigate specific qualitative aspects of GT plants, including the toxicity of glyphosate and GBHs, as well as potential interactions between the GT plant and its herbicide. The project’s findings are summarized below, along with the specific research questions they addressed.

The work commenced by analysing 90 different chemical and biological parameters in 31 individual harvests of soybean obtained from farmers in the state of Iowa (USA), in order to characterise the composition of ‘an average Iowa soybean harvest’ produced in one of three general agriculture methods (industrial GT/Roundup Ready, industrial non-GM, and organic soybeans) [17]. The investigations included multiple toxicity tests of glyphosate and Roundup, as well as multiple life-cycle feeding studies in the versatile aquatic invertebrate model organism *Daphnia magna* (waterflea) to assess the effects of GT soybean in feed formulations. 

## 3. Research Question

### 3.1. Are GT Soybeans Compositionally Equivalent to Their Non-GM Counterparts?

Although early safety studies funded by industry in 1995–1998 concluded that GT soybeans and other herbicide tolerant GM crops were ‘substantially equivalent’ to ordinary varieties, those studies had major flaws, e.g., the plant material was cultivated in artificial conditions in test-fields without spraying with the herbicides that farmers used in commercial production [10,17]. 

Investigating whether commercially-farmed Roundup Ready soy was compositionally equivalent to conventional and organic soybeans, it was a surprise to find that ‘ready-to-market’ Roundup Ready GM soybeans from Iowa, accumulated glyphosate at relatively high levels: on average, 9.0 mg of glyphosate were found per kilo of soybean [17]. In contrast, no glyphosate was found in conventional or organic soybeans grown in the same area and year (which was as expected as those plants would die from glyphosate exposure). Other differences were also found; organic soybean contained significantly more protein, zinc, barium, and several amino acids, and less saturated fat, omega-6 and selenium than the two types of soy from conventional ‘industrial agriculture’ [17]. 

Possibly prompted by those findings, the USDA initiated a separate study of this issue [18], which reported lower residues of glyphosate (approximately 1 mg/kg) and only insignificant compositional differences between Roundup Ready soybeans and conventional soybeans. In contrast, our analysis found that soy from organic, conventional and Roundup Ready soybean could be readily differentiated statistically (with discriminant analysis), based solely on compositional data [17]. Subsequently a new research question arose: would the observed differences in composition translate into significant quality differences when those soybeans were used as feed or food? 

### 3.2. How does the Quality of Feed Made from Roundup Ready Soybeans Compare to Feed from Conventional or Organic Soybeans?

In life-cycle *D. magna* feeding studies the evidence showed that the test organisms performed significantly less well (with lower rates of survival and of reproduction) when fed Roundup Ready soybean as compared to the conventional and organic counterparts [19]. Test animals fed organic soy performed best. The negative effects observed in animals fed Roundup Ready soybean may be explained by (i) the genetic modification itself, or (ii) the residues of glyphosate, or both. To be able to separate those two factors, new experiments were designed to test whether increased levels of glyphosate residues would reduce the quality of soybean-based feed.

### 3.3. Can Adverse Dose-related Effects from Glyphosate Residues be Detected in Consumers?

To investigate whether glyphosate residues in GT soybean had negative effects on the consumer model, *D. magna*, eight different batches of feed were prepared from individual samples of GT soybean, which contained different amounts of glyphosate residues (range: 1.1–15.1 mg/kg, i.e., a factor of approximately 14) [20]. Although all the samples tested complied with the EU and US maximum residue levels (MRLs) of 20 and 40 mg/kg, respectively, it is important to appreciate that residues of other pesticides in food typically are measured at levels of micrograms per kilogramme, i.e., levels that were three orders of magnitude lower. Moreover, the maximum acceptable level for pesticides, including glyphosate, set by EU standards for drinking water is much lower; 0.1 mg/L (Water Framework Directive 2000/60/EC). 

When testing the quality of these different soybean samples as feed, the amount of glyphosate in the soybeans correlated with changes in several life-history traits of *D. magna* test organisms: higher concentrations of glyphosate in the GT soybean-based feed resulted in lower survival, reduced body size and delayed reproduction. The differences observed were relatively small and the explanatory power of the statistics to ascribe the results to the role of glyphosate as a causal factor (*R*^2^) was weak [20]. However, as survival, growth rates and age at maturation all consistently indicated negative effects on the test animals, the data supported the hypothesis that glyphosate residues in the GT soybeans can cause negative effects in consumers. 

### 3.4. Are Glyphosate and Roundup Practically Non-toxic?

Previously, GBHs (Roundup) along with the active ingredient glyphosate (IPA—isopropylamine salt—hereafter referred to as ‘glyphosate’) were claimed to be ‘practically non-toxic’ to animals and humans [21] and as having little or no eco-toxicological effects on the receiving environment [22]. Glyphosate had been reported to have low toxicity in the standard indicator organism *D. magna* [23,24], but that finding was primarily based on toxicity experiments performed by the patent holder of glyphosate (i.e., the Monsanto company) in the 1970s to 1980s and were subsequently presented in widely cited reviews, which reported having found little or no evidence of adverse effects on public health and environmental quality [21,22]. 

Cuhra et al. therefore deemed it relevant to re-test the toxicity of glyphosate in *D. magna* using the same type of assay for acute toxicity (EC_50_—the Effect Concentration, i.e., when a chemical provokes a response such as immobilisation or mortality in 50% of the animals after 48 h) as used by the early industry studies. They used six different clones of *D. magna* and found all of them to be immobilised by concentrations of glyphosate that were 100–300 times lower than the reportedly-safe levels claimed by the industrial stakeholder [25]. The early industry studies indicated that it required 930 mg/L glyphosate to immobilise 50% of the *D. magna* test population within 48 h. This and other similarly high EC_50_ value are still used in available glyphosate Material Safety Data Sheets [26]. Cuhra et al. reported 50% immobilisation/mortality (EC_50_) at concentrations below 10 mg/L [25]. 

Cuhra et al. subsequently performed life-cycle studies on *D. magna* exposed to a range of low concentrations of glyphosate as well as Roundup, and found adverse effects, such as reduced reproduction, at relatively low concentrations (0.45 mg/L), and reduced body size of offspring at 0.05 mg/L, i.e., at lower concentrations than the accepted limit for surface waters in e.g., Australia (1.0 mg/L), the USA (0.7 mg/L) and Switzerland (0.36 mg/L). The experiments indicated that the accepted threshold environmental concentrations of glyphosate in those countries are insufficient to safeguard key freshwater species like water fleas. 

Given those findings, it is misleading to portray glyphosate as ‘practically non-toxic’. Rather, glyphosate should be categorized as ‘moderately toxic’ or ‘toxic’, depending on the toxicity-standard used [25,27]. That conclusion is fully consistent with numerous recent publications, indicating that official toxicity assessments of glyphosate should be revised [28]. 

Moreover, recent results show that agricultural land use with glyphosate and GBH may cause effects of pollution directly on the ecosystem-level, both in freshwater and in the marine environment, by harming primary producers like green and brown algae [29,30]. Ecosystem-level effects can also be mediated by indirect interactions, e.g., through reduction or removal of an aquatic plant due to a low concentration of GBH, which in turn can reduce the abundance of macro-invertebrates (i.e. fish prey) from the same habitat [31].

A recent meta-analysis showed a significant link between exposure to glyphosate and Non-Hodgkin lymphoma [32]. The focus on the putative carcinogenicity of glyphosate has been to the neglect of evidence of adverse effects of various types and end-points, e.g., in soil bacteria [33], and direct and indirect (transgenerational) effects on male sperm quantity and quality in rats [34,35]. In our opinion, the needed re-evaluation of glyphosate toxicity should not be delayed by, or narrowly focused on, the question of whether or not glyphosate should be categorized as a carcinogen [36,37,38]. Moreover, the potential adverse health effects of glyphosate-based herbicides (GBHs) should also consider the full formulations as used on farms. The formulations include multiple adjuvants that can increase the toxicity by orders of magnitude, challenging the correctness of accepted daily intakes [39,40]. That GBHs are produced in about 750 different formulations is complicating the issue further [38]. Moreover, a recent study provided evidence indicating that glyphosate toxicity is much more complex since the chemical may interact synergistically with other stressors, including agrochemicals and heavy metals [41]. 

## 4. The Effective Residue Levels of Glyphosate Are Rising Contrary to Published Recommendations

The Codex Alimentarius Commission’s definition of Good Agricultural Practice (GAP) underlines that ‘…pesticides should be applied in such a way as to leave a residue *which is the smallest amount practicable*’ [42]. Despite such international guidelines and recent research recommendations that glyphosate-based herbicides should be assessed and managed more carefully, i.e., that exposures and consumption should be reduced [43], industrial stakeholders have been successful in gaining acceptance for increasing permitted residue levels of glyphosate. Since the maximum residue level (MRL) vary widely among crops and agencies, the set MRL values seem to be based merely on observed residue contents rather than on toxicological data [44]. 

Two factors that drive increasing residue levels are (i) the prevailing trend for increasing the frequency and concentrations of GBH used directly onto GT crops to combat resistant weeds and (ii) to provide pre-harvest desiccation (the latter applies to both GT and to conventional, but not to organic, crops). These factors increase absorption of the herbicide and augment residue levels in the harvested beans. The number of plant species, in which resistance to glyphosate has been found, increased from two to 43 since 2000 [45], illustrating the challenges posed by the development of herbicide resistance. Paradoxically, the present chemical-based strategy for dealing with resistant weeds is the main factor escalating the problem of resistant (super)weeds and is causing a cascade of agro-ecological consequences, e.g., by creating a strong selection pressure favouring the evolution and dispersal of genes for herbicide tolerance [46]. The evolutionary origins of resistance may come from de novo mutations, from existing genetic variation or from resistant species via hybridisation or horizontal gene transfer [43,47,48]. 

## 5. Application Rates of Glyphosate on Herbicide Tolerant Soybeans: Farmers’ Use *vs* Field Trial Use

**Figure 1:**
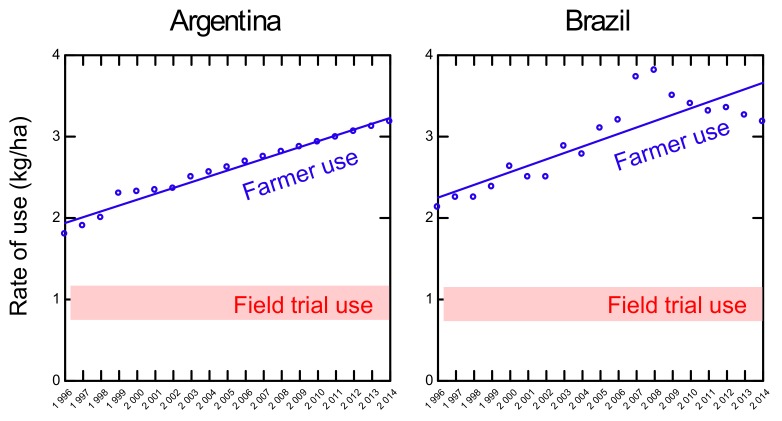
Rate of glyphosate use (active ingredient) in glyphosate-tolerant (GT) soybean production, Brazil and Argentina 1996–2014. Data from Benbrook [3]. Field trial use as recommended by Duke et al. [18] is shown as reference.

Official statistics show that the rates of glyphosate/GBH use in Argentina and Brazil, two of the dominant countries for soy production, have increased significantly from 1996 to 2014 with an almost perfect linear fit (*R*^2^ = 0.98 and 0.91 respectively, *p* < 0.0001, linear regression), reaching 3–4 kg/ha (active ingredient of glyphosate, hereafter termed a.i.) in later years (Figure 1). Thus, these farmer rates are more than twice as high as the recommended doses used in most field trials, i.e., 1.72 kg/ha [18]. 

**Figure 2:**
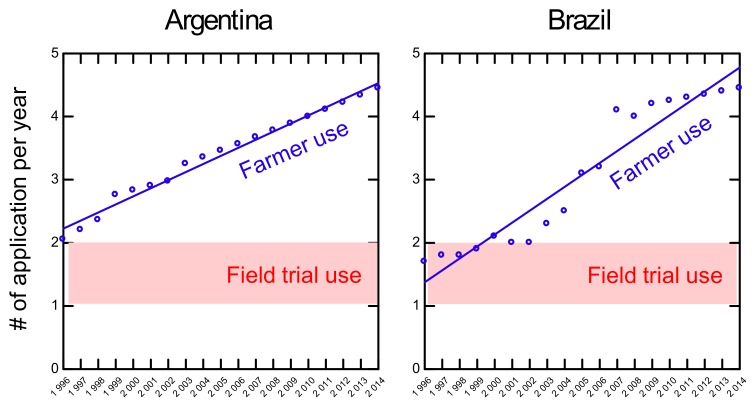
Number of (#) glyphosate/glyphosate-based herbicides (GBH) applications on GT soybeans in Brazil and Argentina 1996–2014, data from Benbrook [3]. Field trial use as recommended by Duke et al. [18] is shown as reference.

The increased spraying of glyphosate/GBH in kg/ha is mirrored by the number of applications per growing season, sprayed by Argentinian and Brazilian farmers. The number of applications show a steady increase, from about two per year in 1996 to more than four applications from 2007 to 2010 and later (Figure 2). Again, the relationship was highly linear (*R*^2^ = 0.97 and 0.74, *p* < 0.0001, linear regression) for Argentina and Brazil, respectively.

Figure 1 and Figure 2 illustrate how the farmers have responded dynamically to increasing problems with resistant weeds. Moreover, these trends show a strong mismatch in spraying regime between field trials compared to the practice of the farmers. The latest applications administered by the farmers, i.e., Sprayings 3 and 4, are most likely performed late in the season. Research has shown that it is the late-season-spraying that increases the residues by a factor of 10 or more in soybean [49]. This is further discussed below. 

These data nonetheless might not represent the actual agricultural practices under commercial conditions in many regions. For example, a study from Argentina reported glyphosate/GBH application rates of no less than 10 kg/ha (a.i.) [50]. Almeida et al. [51] analysed herbicide use in Brazil between 2000 and 2012 and reported an increase to more than 9 kg/ha of glyphosate (a.i.) for soybeans. Based on the data available, Miyazaki et al. [52] concluded that farmers’ use of 6–7 kg/ha (a.i.) seems to be relatively common and even 8–10 kg/ha (a.i.) can be expected under current farming conditions in Argentina, Brazil and Paraguay [52]. 

## 6. Glyphosate Residue Levels and Timing of Spraying

When residues of glyphosate in soybeans are considered, it is important also to include the main breakdown product of glyphosate, namely aminomethylphosphonic acid (AMPA). Hence, in the following, concentrations of glyphosate refer to glyphosate + AMPA. AMPA frequently and widely co-occurs with glyphosate in environmental samples [53] and has a similar toxicity profile to glyphosate [22]. 

**Figure 3:**
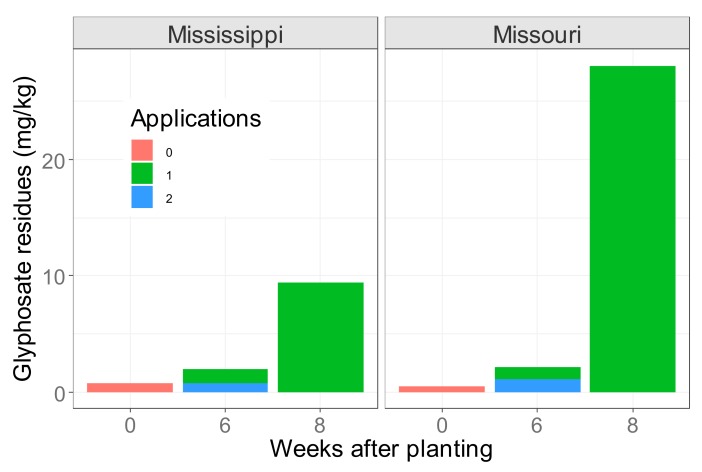
Glyphosate residues (glyphosate + its main breakdown product aminomethylphosphonic acid (AMPA)) in GT soybeans produced in field trials, sprayed with 0, 1 or 2 applications at different time points after planting. Note the crucial importance of late spraying for the residue level found in the soybean grains. Data from Duke et al. [49].

Published data from field trial tests show that the uptake of glyphosate in soybean is highly dependent on the timing of herbicide applications. While plants sprayed once or twice within six weeks after planting resulted in residue levels of less than 3 mg/kg, a single application at eight weeks, i.e., at full bloom of the plant, resulted in a 10–20-fold increase in residual concentrations (i.e., 9.45–28.08 mg of glyphosate per kg, Figure 3) [49]. Those results were consistent across field trial sites in Mississippi and Missouri respectively, and suggest that high residue levels of glyphosate in soybeans come from late season spraying. This makes sense since glyphosate is a systemic herbicide that circulates in the plant and may thus be incorporated into metabolic sinks, such as actively growing soybeans in the treated plants. 

Data from Iowa showed that 60%–70% of the farms had residue levels of 10 mg/kg glyphosate or higher [17], suggesting that spraying late in the season is a widespread practice in that region. Another source of high dietary residue levels of glyphosate, which is unrelated to the use of transgenes, is spraying of non-GT crops just before harvesting, for desiccation, which saves on post-harvest thermal drying costs. This practice is, however, not allowed in organic farming. 

Interestingly, residues of glyphosate have even been found in unsprayed soybean plants, at up to 0.44 mg/kg (Figure 3, left-hand column). The authors argued that herbicide drift was the likely cause of this result, and that “…*with the widespread adoption of GR [GT] soybean and cotton it is difficult to find an experimental site free from glyphosate drift from neighbouring fields*” [49]. A more recent USDA study [18] showed that identically sprayed GT soybean grown in fields with a history of glyphosate/GBH use (over ~15 years) had residues twice as high as plants grown in soil without a history of glyphosate use, indicating that glyphosate accumulates in the soil and can be absorbed into subsequent crops. 

## 7. How Much Glyphosate Enters the Food Chain?

**Figure 4:**
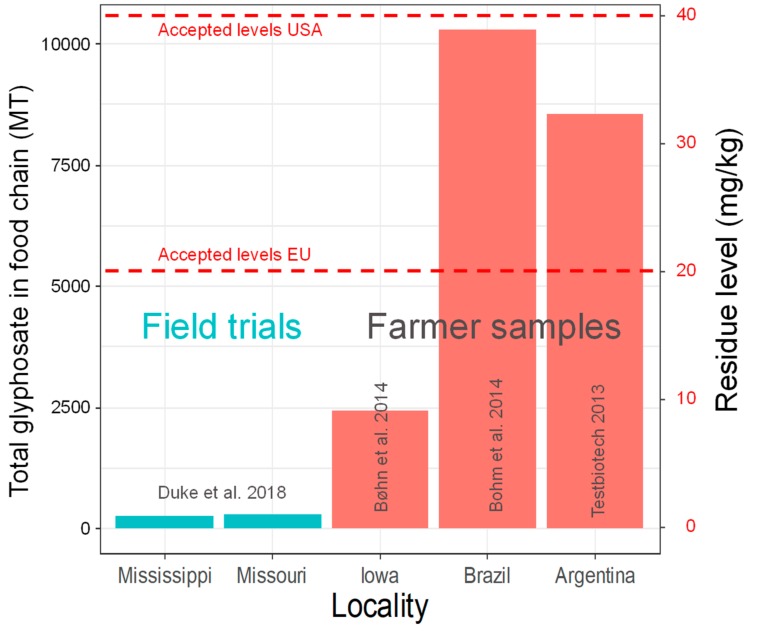
Total estimated amount of glyphosate residues (glyphosate + AMPA) in metric tonnes (MT) that would enter the global food chain (left y-axis), based on calculations using residue-levels per kg (right y-axis) from published data [17,18,54,56]. GT soybeans tested came from corporate field trials (blue) or from farmers (red) in different countries and states in the USA. The total quantity of glyphosate entering the global food chain from GT soybeans was calculated by multiplying the residue levels per kg with the total production of GT soy. Dotted horizontal red lines show maximum accepted residue levels in the EU/Codex and the USA (20 and 40 mg/kg) and how much glyphosate in total would enter the food chain at this level (i.e., an accepted level of ~5000 and ~10000 MT of glyphosate).

Assuming that the glyphosate residue levels found in GT soybeans from Iowa were representative of what is found on the international market, i.e., an average of ~9.0 mg/kg [17], the amount of glyphosate entering the food chain would be 9 g/tonne of soy. This would add up to 2430 tonnes of glyphosate residues from the 270 million MT of GT soy produced globally in the 2016/2017 season (Figure 4). 

Other data from Brazilian and Argentinian farms suggest those estimates are quite conservative. Farm samples from Brazil have shown average residue levels of glyphosate at 38.5 mg/kg [54], i.e., nearly twice as high as the maximum accepted residue level (MRL) as specified by Codex [55] and the EU (Figure 4). In Argentina, average and maximum residue-levels were measured at 31.7 mg/kg (Figure 4) and 72.8 mg/kg, respectively [56]. As discussed above, data from corporate field trials reported far lower residue levels of glyphosate, approximately 1 mg/kg (Figure 4). Still, such concentrations would contribute to an accumulated amount of 270 tonnes of glyphosate into the global food-chains from GT soybeans per year.

## 8. Sprayed Adjuvants to Herbicides Can Be More Toxic But Are Not Monitored

GT soybeans are always sprayed with GBHs, and not only the single active ingredients, such as glyphosate. Apart from the active ingredients, adjuvants (additives and wetting agents) are added to the commercial formulations that farmers use. These adjuvants may enhance the toxicity compared to the single active substance, by a factor of up to 1000 times [39]. For this reason, the use of particularly problematic adjuvants, such as polyethoxylated tallowamine (POEA), is restricted or prohibited in several EU countries. POEA has been shown to be a major component of GBH toxicity, in particular to aquatic organisms such as protozoa, crustaceans, frogs, toads and fish [57].

Nevertheless, the application of POEA is not prohibited in all countries where GT plants are grown. For example, commercial mixtures applied in the fields in Argentina contain typically about 15% of POEA [57], see also [58]. The exact formulations sprayed on the plants are kept secret and treated by corporations and governments as confidential business information. Consequently, it can be expected that GT soybeans imported into the EU from different jurisdictions, such as from South America, have been sprayed with formulations that are not permitted in the receiving countries. Even if an active ingredient such as glyphosate is authorized for use in the EU, detailed investigation of the residues is required for imported food and feed products if the plants were grown under a different regulatory regime/agricultural practice [59]. According to EFSA’s Pesticide Panel, the existing data are in some cases insufficient to assess health risk assessment of GT plants at the stage of consumption [52,60]. 

## 9. A Need for International Coordination of Pesticide Residues in Food and Feed

There is clearly a need for international coordination of policies on pesticide residues. This can be illustrated by the EUs risk assessment of imported GT soybeans. For market authorization of these beans, we highlight the following provisions of the EU Pesticide Regulation:

- Article 29 of Regulation 1107/2009: active substances and synergists have to be approved, the maximum residue levels for the specific agricultural products have to be specified;
- Article 4 of Regulation 1107/2009: pesticides must not have any harmful effects on human or animal health, taking into account known cumulative and synergistic effects;
- Recital 5 of Regulation 396/2005: residues should not be present at levels presenting an unacceptable risk to humans and, where relevant, to animals;
- Recital 10 of Regulation 396/2005: specific MRLs for each pesticide in food and feed products have to be established;
- Recital 26 of Regulation 396/2005: MRLs have to be set for food and feed produced outside the Community if produced by different agricultural practices as regards the use of plant protection products;
- Article 14 of Regulation 396/2005: the presence of pesticide residues arising from sources other than current plant protection uses and their known cumulative and synergistic effects have to be determined; any potential risks to consumers with a high intake and high vulnerability have to be taken into account.

In addition, the EU Commission Implementation Regulation No 503/2013 requires that the field trials with GT plants should compare and test plant products with and without the complementary herbicide being applied. Thus, the data provided for risk assessment should allow scientists to conclude whether the expected agricultural practices influence the tested endpoints. Unfortunately, that provision has not been implemented for GT soybean plants under prevailing risk assessment practices, in the EU or elsewhere, simply because the test materials for risk assessment have not been representative of real-life commercial products from commercial farms. This exemplifies the fact that an essential principle of good science—as realistic test-conditions as possible—has not been applied in the EU’s risk assessment system [61]. 

To make sure a plant product is safe, the materials used for testing need to be representative of the bulk of the materials that are commercially produced, sold and consumed. It is unacceptable that corporate field trials, which are the basis for the safety assessment of GT soybean varieties, have been sprayed according to agricultural practices that differ significantly from contemporary commercial farming. Consequently, those field trials produced unrealistic and irrelevant samples, which may have led risk assessors to have systematically underestimated potential risks.

Importantly, most of the feeding studies to assess the quality of GT soybean (and other GT plants) have used unsprayed test plants [10,62,63]. Those historical errors have unfortunately not been resolved for GT soybean varieties that are currently imported into the EU. For example, the 90 day rat feeding study to test the quality of the triple resistant GT soybean, event DAS-44406-6, called ‘Enlist’ (tolerant to glyphosate, glufosinate ammonium and 2.4-D) was sprayed with lower doses of glyphosate and 2.4-D than recommended for commercial farmers, and was not sprayed at all with glufosinate ammonium [52]. Even when glufosinate ammonium may cause damage to the central nervous system including memory loss in mice [64], and is considered a high-risk chemical to humans [65] (i.e., effects which makes glufosinate ammonium particularly import to include in a mammal feeding study), EFSA accepted the protocol and recently approved this soybean for import.

In the case of ‘Balance Bean’, a triple resistant GT soybean, event FG72 x A5547-127 (tolerant to glyphosate, glufosinate ammonium and isoxaflutole), no feeding study was performed at all as a part of the risk assessment [52]. This event was also recently approved by EFSA for import into the EU.

## 10. Research Material for Independent Testing is not Openly Available

Unfortunately, seeds and samples of GM plant material, including basic data on their composition and quality, are not openly available and this precludes the development of independent research and monitoring strategies [66]. Initially, Bøhn’s research group sent letters and emails, contacting two major industrial biotechnology producers of GT soybean, requesting material for research, albeit with no success. In the interest of society, public health, animal welfare and other research objectives, it is worrying that GT soybean and similar important food and feedstuff from commercial crops are not legally available for independent research and testing [28].

## 11. Conclusions

Research has shown that GT soybeans (i) accumulate herbicides, (ii) have altered nutritional composition, and (iii) cause dose-related adverse effects in feeding studies in a relevant model organism. Moreover, data in the scientific literature show that (i) glyphosate residues are substantially increased when herbicides are applied late in the growing season, (ii) commercial farm samples of GT soybeans contain far higher concentrations of glyphosate compared to GT soybeans from corporate field trials (which were used for safety assessments), and (iii) the total amount of glyphosate introduced into the food chain from GT soybeans adds up to thousands of metric tonnes. The impacts of those residues of glyphosate constitute knowledge gaps that have entailed incomplete evaluations on (i) potential interactions between glyphosate residues and plant composition and (ii) potential negative effects on consumers. These issues are not followed up with proper long-term in vivo feeding studies.
